# High-frequency oscillation ventilation for hypercapnic failure of conventional ventilation in pulmonary acute respiratory distress syndrome

**DOI:** 10.1186/s13054-015-0935-4

**Published:** 2015-05-01

**Authors:** Sigrun Friesecke, Stephanie-Susanne Stecher, Peter Abel

**Affiliations:** Department of Internal Medicine B, Division of Cardiology, Pneumology and Critical Care Medicine, University Medicine Greifswald, Ferdinand-Sauerbruch-Straße, 17475 Greifswald, Germany

## Abstract

**Introduction:**

High-frequency oscillation ventilation (HFOV) is regarded as particularly lung protective. Recently, HFOV has been shown to be not beneficial for acute respiratory distress syndrome (ARDS) patients in general. Due to its special physical effects, it could be beneficial, however, in inhomogeneous ARDS. This study evaluates the effect of HFOV on PaCO_2_ removal in hypercapnic patients with ARDS of pulmonary origin.

**Methods:**

Between October 2010 and June 2014 patients with ARDS of pulmonary origin with PaO_2_/FiO_2_ ratio >60 mmHg, but respiratory acidosis (pH <7.26) under optimized protective ventilation were switched to HFOV, using moderate airway pressure (adopting the mean airway pressure of the prior ventilation). Data from these patients were analyzed retrospectively; PaCO_2_ and pH before, 1 h and 24 h after the start of HFOV were compared.

**Results:**

Twenty-six patients with PaO_2_/FiO_2_ ratio 139 ± 49 and respiratory acidosis (PaCO_2_ 68 ± 12 mmHg) were put on HFOV after 17 ± 22 h of conventional ventilation. Mean airway pressure was 19 cm H_2_O (15 to 28). PaCO_2_ decreased significantly: after 1 hour the mean difference was −14 ± 10 mmHg; *P* <0.01 and after 24 hours −17 ± 12 mmHg; *P* <0.01; n = 24. CO_2_ clearance improved in all but two patients; in those, extracorporeal lung support was initiated. Oxygenation remained unchanged after 1 h and slightly increased after 24 h. No complications related to HFOV were observed. Twenty-two patients improved and could be weaned from HFOV. Twenty patients (77%) were alive on day 30.

**Conclusions:**

HFOV could be a useful alternative in patients with ARDS of pulmonary origin with hypercapnic failure of lung-protective conventional ventilation.

**Electronic supplementary material:**

The online version of this article (doi:10.1186/s13054-015-0935-4) contains supplementary material, which is available to authorized users.

## Introduction

High-frequency oscillation ventilation (HFOV) has been considered to be particularly lung-protective because - compared with conventional ventilation - its physical characteristics may allow better avoidance of both overinflation and recurring alveolar derecruitment [1,2]. Technically, oscillations of 3 to 15 Hz are superimposed onto a constant airway pressure applied via a customary cuffed tracheal tube. The resulting tidal volumes (Vts) often remain below the airway dead space. Effective alveolar ventilation can be achieved nonetheless by physical mechanisms that are explained elsewhere [3-5].

Two recent multicenter studies could not show a survival benefit in adult acute respiratory distress syndrome (ARDS) patients with unselected etiologies of ARDS [6,7]. Therefore, HFOV has so far no place in routine care for ARDS. In special circumstances however, HFOV may be beneficial, mostly as a rescue therapy in oxygenation failure [2,8-10]. A substantial improvement in oxygenation [7,11] and the safety of HFOV have been shown [12,13] even though barotrauma is reported in a substantial number of patients [9,14].

ARDS of pulmonary origin (ARDSpulm) is defined as ARDS caused by an insult primarily affecting the lungs [15]. Compared to ARDS of extrapulmonary origin, in which a hematogeneous insult leads to a diffuse damage with uniform distribution, ARDSpulm shows a distinct computed tomography (CT) morphology with a multifocal and asymmetric involvement [16]. Such inhomogeneously damaged lungs with very different local compliances are exposed to increased stress and strain with conventional ventilation [17]. Complete recruitment often is not possible using conventional ventilation without risk of ventilator-induced lung injury [18]. With conventional ventilation, normally compliant alveoli are exposed to a significant risk of volutrauma due to preferential airflow into these segments [3]. As HFOV is more homogeneous and less dependent on different regional compliances [19], it should be especially advantageous in cases of inhomogeneous pulmonary damage.

We hypothesize a benefit in patients with ARDSpulm and hypercapnic failure of lung-protective conventional ventilation. Ventilatory failure has been suggested as an indication for HFOV [20] but data supporting this alternative are scarce. The main goal is to evaluate CO_2_ removal with HFOV in patients with ARDS with acceptable oxygenation but hypercapnic failure of conventional ventilation. We hypothesize that efficient CO_2_ removal could be achieved with HFOV in these patients.

## Materials and methods

This observational study was done at the medical ICU of the University Medicine of Greifswald between October 2010 and June 2014. The study was approved by the institutional ethics committee at Ernst Moritz Arndt University of Greifswald, who waived the need for informed consent.

Included were all patients with ARDS (novel definition [21], partly adapted retrospectively) of pulmonary origin who received HFOV for respiratory acidosis under lung-protective ventilation with tidal volumes of 6 ml per kg of ideal body weight. HFOV was begun in the case of pH <7.26. If ARDS was accompanied by a cor pulmonale with hemodynamic instability necessitating catecholamines, the threshold for change to HFOV was a milder hypercapnia (pH <7.30). Cor pulmonale was diagnosed by bedside echocardiography (routinely done in every ARDS patient in the initial course) if dilatation of the right ventricle (in apical four-chamber view basal right ventricular diameter ≥ left ventricular diameter) and reduced right ventricular contractility were present (tricuspid annular plane systolic excursion <15 mm). ARDSpulm was diagnosed if a primary pulmonary insult was obvious (as in pneumonia or aspiration) or an inhomogeneous pulmonary CT pattern was evident without a pathogenetic role of systemic inflammation. Patients were excluded from the analysis, if the arterial partial pressure of oxygen/fraction of inspired oxygen (PaO_2_/FiO_2_) ratio was lower than 60 mmHg, necessitating rescue procedures to improve oxygenation. HFOV was not used if therapy was to be limited because of terminal illness or patient’s advance directive, or if HFOV was contraindicated because of severe airway obstruction or intracranial hypertension [20].

Prior to HFOV in all study patients, pressure-controlled conventional ventilation (using a standard ventilator (Evita™, Fa Dräger, Lübeck, Germany)) had been optimized in the following way: determination of the positive end-expiratory pressure (PEEP) optimum for oxygenation by a PEEP trial, adjustment of inspiratory pressure to achieve Vts not larger than 6 ml per kg of ideal body weight, and maximizing respiratory frequency leaving no zero expiratory flow time, but allowing complete expiration. The PEEP trial was performed with a stepwise increase of PEEP level (steps of 2 cmH_2_O) and evaluation of PaO_2_ and arterial partial pressure of carbon dioxide (PaCO_2_) at every step. According to the approach proposed by Rouby to reduce the risk of overinflation in patients with focal loss of aeration, we started with PEEP 5 to 8 cmH_2_O [18]. Patients were sedated according to the local standard operating procedure with propofol and sufentanyl. Muscle relaxants were applied in case of impossibility to achieve patient-ventilator synchrony.

The ICU team has several years’ experience with HFOV. The ventilator used was Vision alpha™, Novalung GmbH, Heilbronn, Germany. Frequency was initially set to a frequency of 5 Hz. A higher start frequency was chosen if a too rapid PaCO_2_ decrease was a concern, that is in case of severe acidosis with pH <7.15. The resulting cycle volume is automatically 90% of the maximal frequency-dependent cycle volume (at 5 Hz 315 ml); it can manually be increased up to 100%. This was done in case of persisting hypercapnia at a frequency of 5 Hz. A cuff leak was not used. Inspiratory to expiratory ratio (I:E) is fixed at 1:1. To optimize lung protection, HFOV frequency was increased in the case of reaching a normal pH and was adjusted to the greatest value that still resulted in an arterial pH <7.29 [22]. Blood gases were checked after the first 30 and 60 minutes of HFOV, then at least two times per shift. Mean airway pressure (mPaw) was set equal to the actual mPaw of the conventional ventilation. As oxygenation was not critically impaired and there was a lower recruitment potential and a higher risk of volutrauma in inhomogeneous ARDS, we refrained from increasing mPaw when changing to HFOV. Sedation was continued in the above mentioned manner. A chest X-ray was taken within 24 hours to rule out overinflation [23].

Outcome parameter was the change in PaCO_2_ after 1 and 24 hours of HFOV, compared to PaCO_2_ pre-HFOV. Statistical analysis was done using paired *t* test. Values are given as mean ± standard deviation. Further data were collected including etiology of respiratory failure, simplified acute physiology score II (SAPS II), duration and setting of pre-HFOV ventilation and of HFOV, 30-day mortality and HFOV-related adverse events. Required minute ventilation (MV) for normal PaCO_2_ (MV x PaCO_2_/40) was calculated additionally as surrogate for dead space [24].

## Results

Eighty-three ARDS patients were treated from October 2010 at this ICU. Twenty-six patients received HFOV because of hypercapnia, most with moderate ARDS. Baseline data are recorded in Table 1.

**Table 1:** **Baseline data and concomitant therapy**

**Number of patients**	**26**
Age (years)	63 ± 16
Male sex	16 (61.5%)
SAPS II	55 ± 15
ARDS	
Severe	6 (23%)
Moderate	19 (73%)
Mild	1(4%)
Diagnosis	
Pneumonia	22 (85%)
Granulomatosis with polyangiitis	1 (3.8%)
Extrinsic allergic alveolitis	1 (3.8%)
Bronchiolitis obliterans with organizing pneumonia	1 (3.8%)
Unknown origin	1 (3.8%)
Ventilation prior to HFOV (all BIPAPassist)	
Duration (hours)	17 ± 22
PEEP (cmH_2_O)	12 [6-20]
Inspiratory pressure (cmH_2_O)	27 [18-36]
Tidal volume (ml)	455 ± 108
Tidal volume (ml/kg of ideal body weight)	6.6 ± 0.9
Minute ventilation (l/min)	9.6 ± 1.8
Frequency	22 ± 6
MV x PaCO_2_/40 (l/min)	15.6 ± 3.0
Proning	6 (23%)

Values are given as number (percentage), mean ± standard deviation, or median [range], respectively; MV x PaCO_2_/40, required minute ventilation for normal PaCO_2_. SAPS II, simplified acute physiology score II; ARDS, acute respiratory distress syndrome; HFOV, high-frequency oscillation ventilation; PEEP, positive end-expiratory pressure; MV, minute ventilation; PaCO_2_, arterial partial pressure of CO_2_.

HFOV was begun 17 ± 22 h after start of mechanical ventilation. Initially, a frequency of 5 to 6 Hz was used and an mPaw between 15 and 28 cmH_2_O (median 18 cmH_2_O). In one patient, pressure had to be reduced because of radiographic signs of overinflation.

After the first hour of HFOV, PaCO_2_ was significantly reduced (−14 mmHg, *P* <0.01; see Table 2). Reduction of PaCO_2_ was seen in all but two patients in whom PaCO_2_ did not improve despite increasing cycle volume. They received additional extracorporeal lung support (iLA™ membrane ventilator, Novalung GmbH, Heilbronn, Germany). These patients were therefore omitted from the 24-hour analysis of PaCO_2_. In the remaining 24 patients, a significant decrease of PaCO_2_ persisted (−17 mmHg after 24 h; *P* <0.01), see Table 2. In another patient, the improved CO_2_ clearance could not be sustained by HFOV, and extracorporeal lung support had to be applied 7 days later.

**Table 2:** **Blood gas analyses before and during high-frequency oscillation ventilation (HFOV)**

	**Before HFOV**	**After 1 h HFOV**	***P***	**After 24 h HFOV**	***P***
Number of patients	26	26		24	
PaCO_2_ (mmHg)	67.6 ± 12.3	53.9 ± 13.7	<0.001	50.4 ± 17.3	<0.001
pH	7.20 ± 0.069	7.28 ± 0.095	<0.001	7.34 ± 0.052	<0.001
PaO_2_/F_i_O_2_ (mmHg)	139 ± 49	125 ± 50	0.17	155 ± 52	0.16

*P* values of paired *t* test. Two patients receiving extracorporeal lung support were omitted from 24-hour analysis. Values are given as mean ± standard deviation. PaCO_2_, arterial partial pressure of carbon dioxide; PaO_2_, arterial partial pressure of oxygen; FiO_2_, fraction of inspired oxygen.

After 24 hours, HFOV frequency had been adjusted to 6 Hz (median, range 5 and 10 Hz); in six patients no increase of frequency had been possible, in eleven patients frequency after 24 h was 6 Hz, in seven patients a frequency of greater than 6 could be chosen. mPaw after 24 h was between 15 and 25 cmH_2_O (median 19 cmH_2_O). Oxygenation remained unchanged after 1 and 24 h (Table 2).

Twenty-two of the 23 patients without extracorporeal lung assist (ECLA) improved and could be switched back to conventional ventilation after 177 (±94) hours. One patient died on HFOV. Twenty patients (77% of the whole cohort) were alive on day 30, 15 (58%) survived to hospital discharge. Six patients died from extrapulmonary reasons, five from nonresolving lung failure. Complications related to HFOV were not observed throughout the study period. In particular, there were no radiographic or clinical signs of barotrauma in any patient.

## Discussion

In ARDSpulm with hypercapnic failure of conventional ventilation, HFOV - implemented early in the course and refraining from increasing mPaw - could effectively remove CO_2_. This was observed in 92% of the patients in this case series. In children, an effective CO_2_ removal has been demonstrated by HFOV [25]. Several studies in adults, however, found PaCO_2_ increasing or unchanged during HFOV [12,13,26-28]. Lubnow *et al*. used an algorithm with initiating HFOV only if pH >7.25 in persistent severe ARDS, and ECLA if <7.25 [29]. The two recent meta-analyses again showed no significant difference in ventilation efficiency or PaCO_2_ [11,30]. So in general, HFOV is not seen as a tool for ventilation failure. In fact, the combination with extracorporeal CO_2_ removal is considered repeatedly [28,31,32]. We used HFOV specifically for CO_2_ removal in patients with ARDSpulm. HFOV has been considered to be a very lung-protective mode. This is expected to be especially relevant for inhomogeneous lung injury in ARDSpulm with consolidation adjacent to normally compliant alveoli, which in this setting may experience cyclic volutrauma with conventional ventilation due to preferential airflow into these segments [3]. HFOV due to its special mode avoids this drawback. Furthermore, in this subset of mainly focal ARDS, there is lower recruitment potential [33] and lower PEEP levels are recommended [18], whereas higher PEEP may propagate an infection or lead to overdistension [34]. Therefore, we decided not to increase the mPaw under conventional ventilation. This is in contrast to the application in recent studies [6,7,12]. Oxygenation did not deteriorate despite leaving mPaw unchanged. Barotrauma did not occur in this setting of HFOV, but has been reported in substantial proportions of patients in previous studies [9,14,22].

Compared to most studies, which did not show an improvement in PaCO_2_, there are some differences: the patients in this study mainly had moderate ARDS; whereas others dealt with severe ARDS [9,27,35]. As oxygenation was acceptable under conventional ventilation, we could refrain from the rather high distension pressures that are frequently inevitable in hypoxemic ARDS. Higher airway pressures have been shown to be associated with lower CO_2_ clearance [9], whereas some data suggest that lower mPaws on HFOV may result in more effective ventilation. In patients with chronic obstructive pulmonary disease (COPD) using mPaw in the same range as we did, a sufficient CO_2_ removal was achieved [36]. Two further studies are in line with our results: Fessler *et al*. demonstrated a sufficient CO_2_ clearance in patients in whom HFOV was initiated partly for ventilation failure [22]. Camparota reported a significant decrease in PaCO_2_ in severe ARDS, predominantly of extrapulmonary origin, with a greater effect in patients with more severe disease and lower respiratory system compliance. They supposed that extrapulmonary origin - implying more recruitment potential - was an important factor for the response to HFOV [10]. In contrast, we found a significant effect in moderate ARDSpulm with more severe pre-HFOV hypercapnia.

Why use HFOV at all after the most recent studies? Two recent studies [6,7] did not show a mortality benefit for HFOV in ARDS, as did two recent meta-analyses [11,30]. These results do not preclude a benefit of this ventilation mode in special patients or circumstances [37]. The use in oxygenation failure remains an indication. Furthermore, HFOV was used successfully in combination with extracorporeal membrane oxygenation (ECMO) in posttraumatic ARDS to recruit a lung with minimal compliance [38]. We used HFOV in patients with respiratory acidosis under protective ventilation. We implemented HFOV early in the course when initial optimizations of ventilation resulted in pH <7.26. This pH threshold for intervention is in accordance to other different studies [29,39] and a consensus article [20].

Initiation of HFOV might not have been the only possibility in the reported patients. Those with pH ≥7.20 under ventilation with Vt 6 ml/kg could have remained on conventional ventilation. There are, however, concerns regarding adverse effects of hypercapnia [40,41]. Furthermore, in focal ARDS even a Vt of 6 ml per kg may result in hyperinflation [34,42]. As Vt was already at the higher margin, we aimed to minimize hypercapnia with an alternative ventilation technique, which is supposed to be more protective [1]. With pH <7.20, conventional ventilation could have been complemented by extracorporeal CO_2_ elimination, which was regarded as more invasive and therefore reserved as a fallback solution. We actually used ECLA when HFOV failed to improve CO_2_ clearance. In a similar way, Lubnow *et al*. treated their patients with ARDS with initial HFOV [29].

There are several limitations of this study: this is a retrospective analysis of a small sample size. With HFOV far away from being a standard procedure, we report our experience in a clearly defined subgroup of patients with ARDS, including all consecutive ones in a definite period. Furthermore, we did not examine parameters of pulmonary stress or inflammation, so we cannot draw conclusions regarding benefit of this alternative strategy.

## Conclusions

In a high percentage of ARDSpulm with hypercapnic failure of lung-protective conventional ventilation, we found effective CO_2_ removal by HFOV instituted early in the course and using distending pressures equal to that of the preceding conventional ventilation. These patients may benefit from HFOV as an especially lung-protective strategy. The role of HFOV in hypercapnia management facing different options, including extracorporeal techniques, remains to be determined.

## Key messages

- Protective ventilation in ARDS may lead to severe respiratory acidosis necessitating an alternative or additional approach.
- With HFOV a more effective CO_2_ elimination may be achievable compared to conventional protective ventilation in ARDSpulm.
- HFOV may be an alternative in hypercapnic failure of protective ventilation in patients with ARDS.

## Abbreviations

ARDS: acute respiratory distress syndrome
ARDSpulm: ARDS of pulmonary origin
COPD: chronic obstructive pulmonary disease
CT: computed tomography
ECLA: extracorporeal lung assist
ECMO: extracorporeal membrane oxygenation
FiO_2_: fraction of inspired oxygen
HFOV: high-frequency oscillation ventilation
I:E: inspiratory to expiratory ratio
mPaw: mean airway pressure
MV: minute ventilation
PaCO_2_: arterial partial pressure of carbon dioxide
PaO_2_: arterial partial pressure of oxygen
PEEP: positive end-expiratory pressure
SAPS II: simplified acute physiology score II
Vt: tidal volume
